# Habitat radiomics and deep learning on gadoxetic acid-enhanced MRI for noninvasive assessment of CK19 expression and recurrence-free survival in hepatocellular carcinoma

**DOI:** 10.3389/fonc.2025.1684264

**Published:** 2025-11-10

**Authors:** Weihao Chen, Jingcheng Hu, Mingzhan Du, Tao Zhang, Chunyan Gu, Qian Wu, Yanfen Fan, Ximing Wang, Yixing Yu, Chunhong Hu

**Affiliations:** 1 Department of Radiology, First Affiliated Hospital of Soochow University, Suzhou, China; 2 Department of Endocrinology, First Affiliated Hospital of Soochow University, Suzhou, China; 3 Department of Pathology, First Affiliated Hospital of Soochow University, Suzhou, China; 4 Department of Radiology, Nantong Third Hospital Affiliated to Nantong University, The Third People’s Hospital of Nantong, Nantong, China; 5 Department of Pathology, Nantong Third Hospital Affiliated to Nantong University, The Third People’s Hospital of Nantong, Nantong, China

**Keywords:** magnetic resonance imaging, deep learning, habitat radiomics, cytokeratin 19, hepatocellular carcinoma

## Abstract

**Objectives:**

To develop a non-invasive model for the preoperative prediction of Cytokeratin 19 (CK19) expression in hepatocellular carcinoma (HCC) based on clinical, radiologic, habitat radiomics, and deep learning features using gadoxetic acid-enhanced MRI, and to assess its utility for RFS risk stratification.

**Methods:**

In this retrospective study, 539 patients with HCC from two hospitals were divided into training (n = 266), internal (n = 114), and external (n = 159) test sets. Univariable and multivariable logistic regression analyses were conducted on clinical and radiologic features to develop a clinical-radiologic model. Habitat radiomics and deep learning (DL) features were extracted and selected to develop the Habitat and DL models, respectively. The DL-HR nomogram model incorporating clinical, radiologic, habitat radiomics, and deep learning features was developed and evaluated. The Kaplan-Meier survival analysis assessed recurrence-free survival (RFS) in the CK19-positive (CK19+) and CK19-negative (CK19-) patients.

**Results:**

AFP level and arterial phase (AP) enhancement were identified as independent predictors of CK19 expression. The DL-HR nomogram model showed superior performance compared to the clinical-radiologic model in both internal and external test sets (all P < 0.05). The AUCs of the DL-HR nomogram and clinical-radiologic models were 0.794 [95% CI: 0.708-0.864] vs. 0.615 [95% CI: 0.520-0.705] for the internal test set and 0.744 [95% CI: 0.669-0.810] vs. 0.600 [95% CI: 0.520-0.677] for the external test set, respectively. RFS was significantly different between the DL-HR nomogram model-predicted CK19+ and CK19- HCC patients across all sets (all P < 0.05).

**Conclusions:**

The DL-HR nomogram model integrating clinical, radiologic, habitat radiomics, and deep learning features effectively predicted the CK19 expression and served as an effective tool for RFS risk stratification in HCC.

## 1 Introduction

Cytokeratin 19 (CK19) is well acknowledged as a marker of biliary/progenitor cells and tumor stem cells and represent a vital marker of the proliferative subtype in Hepatocellular Carcinoma (HCC) (1). Clinically, CK19 is expressed in approximately 10-30% of the HCCs (2). Patients with CK19-positive (CK19+) HCC demonstrate poorer prognosis, reduced overall survival (OS) and recurrence-free survival (RFS), and higher recurrence rates, compared with CK19-negative (CK19-) HCC patients (3, 4). Therefore, accurate prediction of CK19 holds crucial importance for early clinical decision-making.

Currently, the identification of CK19 in HCC relies on pathological immunohistochemical assessment (5). Magnetic resonance imaging (MRI) provides a valuable and non-invasive method for preoperative pathologic evaluation of HCC (6). Despite this, radiologists still face challenges in accurately identifying HCC subtypes from original MRI images. In recent years, deep learning (DL) algorithms have gained prominence for their ability to directly mine the complex features of visual information from imaging data, and are widely used in the fields of deep feature extraction of medical images, tumor recognition, differential diagnosis, and grading (7–10). Habitat radiomics analysis is an emerging method applied to various diseases. Tumor subregions, or “habitats,” represent clusters of tissue with similar properties, providing valuable insights into tumor phenotypes and the tumor microenvironment. Habitat analysis can provide tumor-specific multidimensional features, thereby improving predictive performance (11). Previous studies have shown that radiomics analysis of MRI models is helpful to assess CK19 expression (12–14). To our knowledge, few studies have combined habitat radiomics and deep learning analyses for predicting CK19 expression.

Therefore, this study aimed to develop and validate a predictive model for CK19 expression and RFS in HCC by integrating clinical, radiologic, habitat radiomics, and deep learning features derived from gadoxetic acid-enhanced MRI.

## 2 Materials and methods

### 2.1 Patients

The institutional ethics review boards in our medical centers approved this study and waived informed consent requirements due to the study’s retrospective nature. Patients with HCC who underwent gadoxetic acid-enhanced MRI examinations at two medical centers between June 2016 and June 2024 were enrolled. Inclusion criteria were as follows: (a) no less than 18 years of age, (b) histologically confirmed HCC and available CK19 immunohistochemical staining data, (c) underwent gadoxetic acid-enhanced MRI within 20 days before hepatic resection, and (d) no prior treatment for HCC before the MRI examination. Exclusion criteria were as follows: (a) Images with poor clarity, (b) Incomplete clinical data or pathologic examination, and (c) mixed liver malignancies other than HCC, including combined hepatocellular-cholangiocarcinoma (cHCC-CCA) and intrahepatic cholangiocarcinoma (ICC). In patients with multiple HCCs, the largest tumor was selected as the main object.

A total of 539 patients were included in this study, consisting of 117 CK19+ and 422 CK19- patients. Patients from institute 1 (n = 380) were randomly assigned to the training set (n = 266; 55 CK19+ and 211 CK19-) and internal test set (n = 114; 28 CK19+ and 86 CK19-) at a ratio of 7:3. Additionally, patients from institute 2 served as the external test set (n = 159; 34 CK19+ HCC and 125 CK19-).

**Figure 1:**
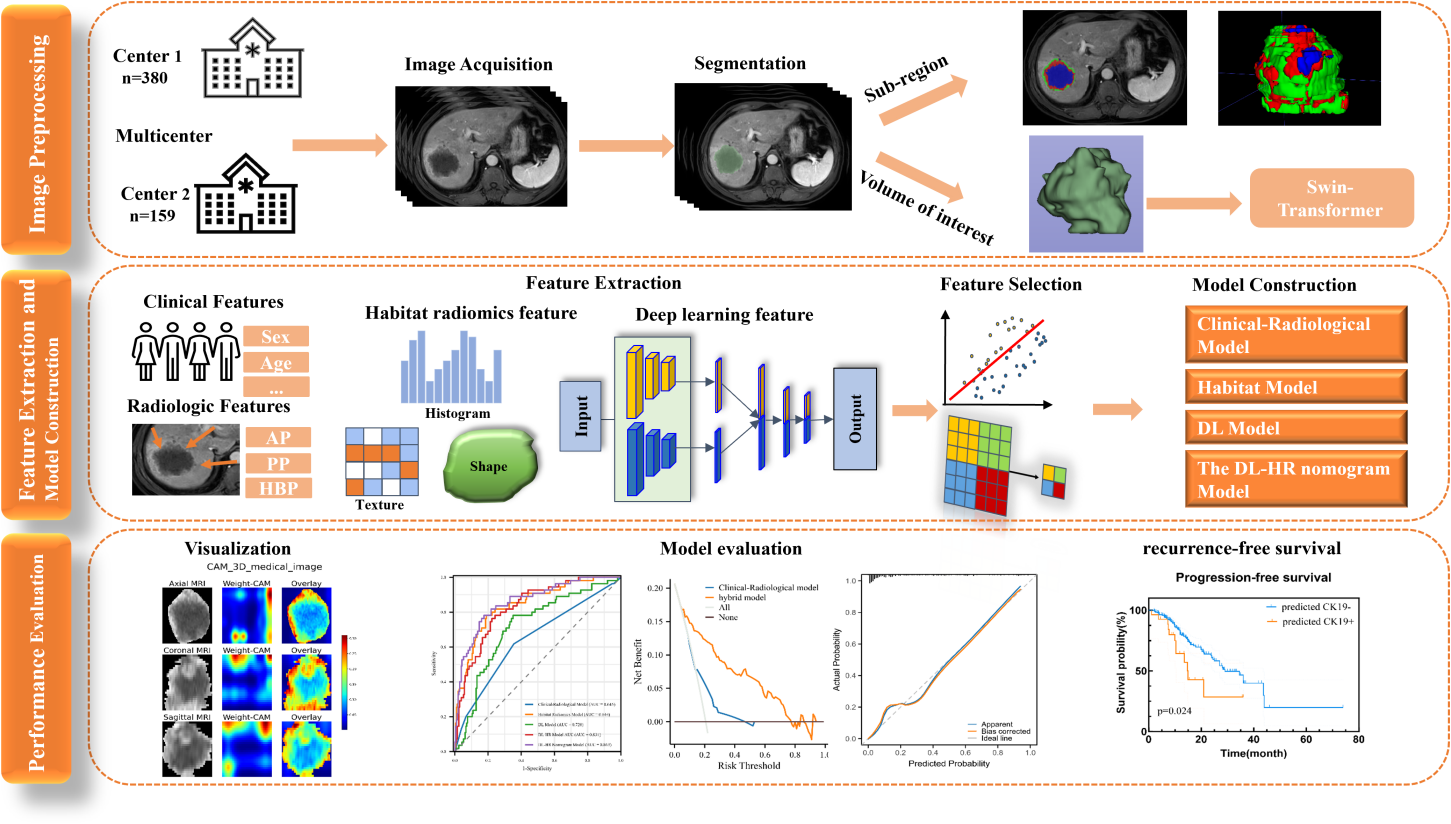
Workflow of this study. Workflow of this study for Predicting CK19 Expression and Recurrence-Free Survival in Hepatocellular Carcinoma. HCC, hepatocellular carcinoma. Cytokeratin 19, CK19. RFS, recurrence-free survival.

Baseline clinical information was obtained from electronic medical records, including age, sex, viral hepatitis status, liver cirrhosis, alpha-fetoprotein (AFP), alanine aminotransferase (ALT), aspartate aminotransferase (AST), gamma-glutamyl transferase (GGT), tumor maximum diameter, and tumor number. The definition of tumor maximum diameter is the maximum length of the tumor in the axial plane. The workflow of this study was shown in **Figure 1**.

### 2.2 Histopathology and immunohistochemistry

CK19 expression data were collected from pathology reports. Pathologic evaluations were performed independently and qualitatively by two pathologists (C.Y.G., Reader 1 and M.Z.D., Reader 2, with 8 and 10 years of experience in pathology, respectively). Disagreements were resolved through consensus discussion. CK19 positivity was defined as membranous or cytoplasmic immunoreactivity presented in≥ 5% of tumor cells (4).

### 2.3 MRI acquisition and imaging analysis

Two 3.0-T MRI scanners (Siemens Magnetom Skyra and GE Revolution) were used to acquire MR images. The sequences employed T1-weighted in-phase, T2-weighted imaging, diffusion-weighted imaging, as well as contrast-enhanced T1WI imaging using gadoxetic acid (Bayer Healthcare, Germany). The detailed imaging protocols are shown in **Supplementary material Table S1**.

Radiologic characteristics were evaluated by two radiologists (W.H.C., Reader 1, and Y.X.Y., Reader 2, with 2 and 11 years of experience in liver imaging), respectively, in consensus to avoid subjectivity. Although the Readers were aware that all patients had HCC, they remained blinded to the clinical, laboratory, and histologic data. The following 16 MRI imaging features were evaluated: Shape of tumor, Arterial phase (AP) enhancement, Arterial peritumoral enhancement, Capsule, Washout, Delayed enhancement, LI-RADSv2018 category, Intratumoral artery, Fat in mass, Blood production in mass, Substantial necrosis, Hepatobiliary phase (HBP) signal intensity, peritumoral hepatobiliary phase hypointensity, and Diffusion-weighted imaging (DWI) signal intensity.

### 2.4 Follow−up

Postoperative follow-up was performed using abdominal MRI at 3-6month intervals. Tumor recurrence was defined through typical imaging features at MRI or pathological results. RFS was defined as the time from the surgery date to the date of the first recurrence, metastasis, or last follow-up. The deadline for the follow-up period was February 10, 2025.

### 2.5 Habitat radiomics and deep learning features extraction

Three radiologists, each with two years of experience, used 3D Slicer Software(http://www.slicer.org) for segmentation. Three-dimensional masks (volumes of interest) of the tumors were manually segmented on a slice-by-slice basis in arterial phase (AP), portal venous phase (PP), and hepatobiliary phase (HBP) images. All VOIs were reviewed by a senior radiologist (Y.X.Y., 11 years of experience) for quality control. These tumor VOIs were then used for the subsequent extraction of habitat radiomics and deep learning features.

All MRI images underwent the following preprocessing steps: Bias field correction was performed using the N4 algorithm to mitigate intensity nonuniformity. Intensity normalization with a scaling factor of 100 was applied to reduce signal variability across scanners and to standardize voxel intensity distributions prior to radiomic feature extraction. Voxel resampling was conducted to achieve an isotropic resolution of 2×2×2mm³, ensuring consistency in spatial resolution across all images.

3D Swin Transformer models were developed for feature extraction from AP, PP, and HBP images, respectively. These models were based on the “swin3d_s” architecture available in the torchvision models. video module of PyTorch 2.1.0 (https://github.com/pytorch/pytorch). Several techniques were implemented to mitigate overfitting, including data augmentation protocols (Resized, ScaleIntensityRanged, RandFlipd, and RandAffined) and learning rate decay algorithms. In this study, a batch size of 16 and an initial learning rate of 0.0001 were used, with the learning rate decaying by a factor of 0.1 every 7 epochs. To handle class imbalance, we employed focal loss as the loss function. The Adam optimizer was selected due to its ability to adjust the learning rate automatically. Training continued for 200–500 epochs. The finalized weights were leveraged to extract robust feature representations from the imaging data. Ultimately, 768 deep learning features were obtained from each VOI.

The unsupervised K-means clustering was applied to generate similar subregions within tumor voxels using cluster numbers (k) ranging from 2 to 10 to control clustering resolution. The optimal number of clusters was determined to be 3 based on the highest Calinski-Harabasz (CH) index value. After clustering, same colors were assigned to pixels in the same cluster, creating subregion maps that provided visually intuitive representation and served as imaging biomarkers for quantifying intratumor heterogeneity. Radiomics features were extracted from VOIs using the PyRadiomics package (http://www.radiomics.io/pyradiomics.html). Radiomics features can be calculated on the original or pre-processed images using the wavelet and Laplacian of Gaussian (LoG) filters (sigma = 2.0, 3.0, 4.0, 5.0). Feature computation was performed at resampling voxel dimensions of 2×2×2mm³ and an intensity bin width of 5. Radiomics features included first-order statistics and texture metrics derived from gray level co-occurrence matrix, gray level dependence matrix, gray level size zone matrix, and neighboring gray tone difference matrix. Lastly, each cluster contained 1132 features, totaling 3396 habitat radiomic features were obtained from each tumor VOI.

### 2.6 Feature selection and models development

On the basis of training set, univariable logistic regression analyses identified clinical and radiologic features associated with CK19 expression. The remaining features underwent multivariable logistic regression to select the independent predictors. The variables associated with CK19 in univariable analysis (p < 0.1) were used to develop the clinical-radiologic model using stepwise backward logistic regression.

Habitat radiomics and deep learning feature analysis were performed using FAE software version 0.5.6 (https://github.com/salan668/FAE), a PyRadiomics-based analytical platform. Data preprocessing involved synthetic minority over-sampling algorithm (SMOTE) for class imbalance correction and Z-score normalization for feature standardization. Features with Pearson correlation coefficients (PCC) >0.8 were considered highly correlated. When two features were highly correlated, the feature with a stronger correlation with CK19 expression was retained to reduce redundancy and ensure statistical independence. Feature selection utilized recursive feature elimination (RFE) and analysis of variance (ANOVA) testing. Logistic regression (LR) and support vector machine (SVM) algorithms were used for model development. The Habitat and DL model scores for CK19 were calculated based on the mean predicted probabilities from the best model.

Model performance was evaluated using the area under the receiver operating characteristic curve (AUC). The optimal habitat radiomics and DL features were integrated with selected clinical and radiologic features through multivariable logistic regression to construct the DL-HR nomogram model. Highly collinear features were removed using variance inflation factor (VIF) analysis.

### 2.7 Statistical analysis

Statistical analysis was conducted using SPSS Statistics (version 24.0) and MedCalc software. The Shapiro-Wilk test assessed normality. Normally distributed continuous variables are expressed as mean and standard deviation, while non-normally distributed continuous variables are presented as medians with interquartile range (IQRs). Categorical variables are presented as frequencies with percentages. Independent sample t-tests or Mann-Whitney U tests were used for quantitative variables and chi-square tests for categorical variables. Univariable and multivariable logistic regression analyses were conducted to identify the independent predictors, using a stepwise backward selection.

Kaplan-Meier analysis with the log-rank test was used to evaluate RFS outcomes. The correlation heatmap, decision curve analysis (DCA), and nomogram were generated using the “corrplot”, “rmda”, and “rms” packages, respectively, in R software (version 4.4.2). Model performance was evaluated using Receiver operating characteristic (ROC) curves, AUC, sensitivity, and specificity. The DeLong test was used for model comparison. P-value < 0.05 (two-tailed) was considered statistically significant.

## 3 Results

### 3.1 Baseline characteristics

A total of 539 patients (mean age [± SD], 59.9 years ± 10.2; 412 male, 127 female) were included. Most patients had hepatitis virus infection (414 of 539 [76.8%]). Clinical and radiologic characteristics showed no statistically significant differences between the training and internal test sets, except for washout. The distribution of CK19 expression status remained consistent across all sets: training set (CK19+ n = 55 [20.7%]; CK19- n = 211 [79.3%]), internal test set (CK19+ n = 28 [24.6%]; CK19- n = 86 [75.4%]), and external test set (CK19+ n = 34 [21.4%]; CK19- n = 125 [78.6%]). CK19 expression was similarly distributed between the training and internal test set (p = 0.401, χ² = 0.705) or external test set (p = 0.862, χ² = 0.030). All baseline characteristics are detailed in **Tables 1** and **2**.

**Table 1:** Clinical characteristics of patients with HCC.

Characteristics	Training set (n = 266)		Internal test set (n = 114)		External test set (n = 159)		P^#^	P^*^
	Ck19 (–) (n=211)	Ck19(+) (n=55)	Ck19 (–) (n=86)	Ck19(+) (n=28)	Ck19 (–) (n=125)	Ck19(+) (n=34)		
Sex^a^							0.211	0.251
Male	164(77.7)	40(72.7)	68(79.1)	26(92.3)	89(71.2)	25(73.5)		
Female	47(22.3)	15(27.3)	18(20.9)	2(7.7)	36(28.8)	9(26.5)		
Age^b^(years)	61.0 ± 10.0	57.2 ± 11.9	61.9 ± 10.9	56.8 ± 9.5	59.0 ± 9.1	58.7 ± 10.0	0.740	0.210
HBV^a^							0.180	<0.001
negative	62(29.4)	19(34.5)	24(27.9)	3(10.7)	14(11.2)	3(8.8)		
positive	149(70.6)	36(65.5)	62(72.1)	25(89.3)	111(88.8)	31(91.2)		
Cirrhosis^a^							0.194	<0.001
negative	110(52.1)	33(60.0)	39(45.3)	14 (50)	45(36.0)	12(35.3)		
positive	101(47.9)	22(40.0)	47(54.7)	14 (50)	80(64.0)	22(64.7)		
ALT^a^(U/L)							0.810	0.750
≤50	167(79.1)	39(70.9)	70(81.4)	17(60.7)	91(72.8)	30(88.2)		
>50	44(20.9)	16(29.1)	16(18.6)	11(39.3)	34(28.2)	4(11.8)		
AST^a^(U/L)							0.961	0.011
≤40	149(70.6)	37(67.3)	61(70.9)	19(67.9)	68(54.4)	24(70.6)		
>40	62(29.4)	18(32.7)	25(29.1)	9(32.1)	57(45.6)	10(29.4)		
GGT^a^(U/L)							0.653	0.292
≤100	118(55.9)	27(49.1)	51(59.3)	14(50.0)	75(60.0)	20(58.8)		
>100	93(44.1)	28(50.9)	35(40.7)	14(50.0)	50(40.0)	14(41.2)
AFP^a^(μg/L)							0.449	0.003
≤100	140(66.4)	25(45.5)	55(64.0)	11(39.3)	100(80.0)	21(61.8)		
>100	71(33.6)	30(54.5)	31(36.0)	17(60.7)	25(20.0)	13(38.2)		

^a^ Data are numbers of patients, with percentages in parentheses. ^b^ Data are means ± SDs.P^#^ < 0.05 indicates a statistically significant difference between the training and internal test; P^*^ < 0.05 indicates a statistically significant difference between the training and external test set. HBV, hepatitis B virus; ALT, alanine aminotransferase; AST, aspartate amino-transferase; GGT, γ-glutamyl transferase; AFP, α-fetoprotein; CK19, Cytokeratin 19.

**Table 2:** Radiologic characteristics of patients with HCC.

Characteristics	Training set (n = 266)		Internal test set (n = 114)		External test set (n = 159)		P^#^	P^*^
	Ck19 (–) (n=211)	Ck19(+) (n=55)	Ck19 (–) (n=86)	Ck19(+) (n=28)	Ck19 (–) (n=125)	Ck19(+) (n=34)		
Tumor margin^a^							0.649	0.536
smooth	139(65.9)	39(70.9)	60(69.8)	19(67.9)	90(72.0)	21(61.8)		
non-smooth	72(34.1)	16(29.1)	26(30.2)	9(32.1)	35(28.0)	13(38.2)		
Tumor number^a^							0.611	0.003
1	146(69.2)	36(65.5)	61(70.9)	20(71.4)	103(82.4)	27(79.4)		
≥2	65(30.8)	19(34.5)	25(29.1)	8(28.6)	22(17.6)	7(20.6)		
Diameter(cm)^c^	4.7(2.9-7.5)	3.9(2.7-6.4)	4.2(2.6-6.7)	4.1(2.5-8.0)	2.7(1.7-4.2)	2.4(1.5-4.2)	0.516	<0.001
AP enhancement^a^							0.821	0.912
negative	11(5.2)	2(3.6)	6(7.0)	1(3.6)	8(6.4)	1(2.9)		
non-rim	186(88.2)	42(76.4)	72(83.7)	23(82.1)	106(84.8)	28(82.4)		
rim	14(6.6)	11(20.0)	8(9.3)	4(14.3)	11(8.8)	5(14.7)		
AP peritumor enhancement^a^							0.567	0.347
negative	178(84.4)	42(76.4)	75(87.2)	22(78.6)	110(88.0)	27(79.4)		
positive	33(15.6)	13(23.6)	11(12.8)	6(21.4)	15(12.0)	7(20.6)		
capsule^a^							0.051	<0.001
without capsule	76(36.0)	25(45.5)	29(33.7)	8(28.6)	55(44.0)	14(41.2)		
enhancing capsule	108(51.2)	25(45.5)	45(52.3)	16(57.1)	69(55.2)	17(50.0)		
non-enhancing capsule	27(12.8)	5(9.1)	12(14.0)	4(14.3)	1(0.8)	3(8.8)		
Washout^a^							<0.001	<0.001
without	64(30.3)	14(25.5)	23(26.7)	11(39.3)	20(16.0)	6(17.6)		
nonperipheral washout	140(66.4)	35(63.6)	59(68.6)	13(46.4)	99(79.2)	24(70.6)		
peripheral washout	7(3.3)	6(10.9)	4(4.7)	4(14.3)	6(4.8)	4(11.8)		
Delayed enhancement^a^							0.790	0.041
negative	150(71.1)	41(74.5)	63(73.3)	18(64.3)	105(84.0)	26(76.5)		
positive	61(28.9)	14(25.5)	23(26.7)	10(35.7)	20(16.0)	8(23.5)		
LI-RADS category^a^							0.837	<0.001
LR-3	11(5.2)	2(3.6)	7(8.1)	2(7.1)	12(9.6)	4(11.8)		
LR-4	32(15.2)	10(18.2)	13(15.1)	5(17.9)	9(7.2)	6(17.6)		
LR-5	149(70.6)	30(54.5)	57(66.3)	16(57.1)	104(83.2)	24(70.6)		
M	16(7.6)	13(23.6)	8(9.3)	5(17.9)	0(0.0)	0(0.0)		
TIV	3(1.4)	0(0.0)	1(1.2)	0(0.0)	0(0.0)	0(0.0)		
Intratumoral artery^a^							0.250	<0.001
negative	114(54.0)	30(54.1)	53(61.6)	16(57.1)	87(69.6)	25(73.5)		
positive	97(46.0)	25(45.5)	33(38.4)	12(42.9)	38(30.4)	9(26.5)		
Fat in mass^a^							0.687	0.601
negative	164(77.7)	41(74.5)	69(80.2)	21(75.0)	96(76.8)	30(88.2)		
positive	47(22.3)	14(25.5)	17(19.8)	7(25.0)	29(23.2)	4(11.8)		
Blood product in mass^a^							0.598	0.003
negative	155(73.5)	46(83.6)	67(77.9)	22(78.6)	109(87.2)	30(88.2)		
positive	56(26.5)	9(16.4)	19(22.1)	6(21.4)	16(12.8)	4(11.8)		
necrosis^a^							0.622	0.008
without necrosis	121(57.3)	33(60.0)	53(61.6)	16(27.1)	89(71.2)	27(79.4)		
Patch necrosis	69(32.7)	16(29.1)	26(30.2)	11(39.3)	29(23.2)	4(11.8)		
Mass necrosis	21(10.0)	6(10.9)	7(8.1)	1(3.6)	7(5.6)	3(8.8)		
HBP signal intensity^a^							0.281	0.021
low	191(90.5)	54(98.2)	84(97.7)	26(92.9)	124(99.2)	32(94.1)		
equal	11(5.2)	1(1.8)	1(1.2)	1(3.6)	1(0.8)	2(5.9)		
high	9(4.3)	0(0.0)	1(1.2)	1(3.6)	0(0.0)	0(0.0)		
HBP peritumoral hypointensity^a^							0.209	0.021
negative	177(83.9)	40(72.7)	75(87.2)	24(85.7)	113(90.4)	30(88.2)		
positive	34(16.1)	15(27.3)	11(12.8)	4(14.3)	12(9.6)	4(11.8)		
DWI signal intensity^a^							0.422	0.537
slightly high	183(86.7)	51(92.7)	74(86.0)	23(82.1)	104(83.2)	30(88.2)		
high	18(8.5)	4(7.3)	11(12.8)	3(10.7)	15(12.0)	3(8.8)		
equal	10(4.7)	0(0.0)	1(1.2)	2(7.1)	6(4.8)	1(2.9)		

^a^ data are numbers of patients, with percentages in parentheses. ^b^ Data are means ± SDs. ^c^ Data are medians, with IQRs in parentheses. P^#^ < 0.05 indicates a statistically significant difference between the training and internal test; P^*^ < 0.05 indicates a statistically significant difference between the training and external test set.

AP, arterial phase; HBP, hepatobiliary phase; DWI, Diffusion weighted imaging; LI-RADS, Liver Imaging Reporting and Data System; TIV, tumor in vein.

### 3.2 Performance evaluation of prediction models

#### 3.2.1 Performance of clinical-radiologic model

Univariable analysis showed that five clinical and radiologic factors were associated with the expression of CK19 in the training set, including age, AFP, AP enhancement, washout, and peritumoral hepatobiliary phase hypointensity. Multivariable analysis revealed AFP level > 100 μg/L (OR = 2.519 [95% CI:1.129-5.617]; p = 0.024), AP enhancement (OR = 2.996 [95% CI: 0.976-9.194]; p = 0.055) as independent predictors of CK19 expression. These factors were used to construct the clinical-radiologic model (**Table 3**).

**Table 3:** Explore the predictors with binary logistic regression analysis.

Characteristics	Univariable			Multivariable		
	β	P	OR(95%CI)	β	P	OR(95%CI)
age	-0.034	0.018	0.966(0.939-0.994)		NA	NA
AFP	0.861	0.005	2.366(1.259-4.323)	0.924	0.024	2.519(1.129-5.617)
AP hyper enhancement	1.030	0.009	2.801(1.286-6.101)	1.097	0.055	2.996(0.976-9.194)
washout	0.659	0.030	1.933(1.067-3.503)		NA	NA
peritumoral hepatobiliary phase hypointensity	0.669	0.060	1.952(0.972-3.922)		NA	NA
Habitat	1.437	<0.001	4.208(2.846-6.222)	1.577	<0.001	4.839(3.073-7.621)
DL	0.842	<0.001	2.320(1.616-3.330)	1.070	<0.001	2.916(1.818-4.677)

OR, odds ratio; AFP, α-fetoprotein; AP, arterial phase; HBP, hepatobiliary phase; Habitat, Habitat radiomic; DL, deep learning; NA, not applicable.

The clinical-radiologic model achieved AUCs of 0.645 [95% CI: 0.584-0.702], 0.615 [95% CI: 0.520-0.705] and 0.600 [95% CI: 0.520-0.677] in the training, internal test and external test sets, respectively, for predicting CK19 expression (**Table 4**).

**Table 4:** Comparison of performance of DL-HR nomogram and clinical-radiologic model.

Model and metric	DL-HR nomogram model	Clinical-radiologic model	P	Z
Training Set (n =266)				
Sensitivity	0.782	0.618		
Specificity	0.825	0.645		
AUC (95%CI)	0.863(0.815-0.902)	0.645(0.584-0.702)	<0.001	4.159
Internal test Set (n = 114)				
Sensitivity	0.750	0.607		
Specificity	0.756	0.616		
AUC (95%CI)	0.794(0.708-0.864)	0.615(0.520-0.705)	0.008	2.665
External test Set (n =159)				
Sensitivity	0.618	0.441		
Specificity	0.872	0.744		
AUC (95%CI)	0.744(0.669-0.810)	0.600(0.520-0.677)	0.017	2.382

Sensitivity and specificity are percentages, AUC = area under the receiver operating characteristic curve. 95% CI = 95% confidence interval,

* The DL-HR nomogram includes AFP levels (>100 ng/mL), arterial enhancement, and predictive score of Habitat radiomics and deep learning models.

P and Z value was calculated with the DeLong test.

#### 3.2.2 Performance of habitat, DL and habitat-DL models

Four Habitat, four DL, and four Habitat-DL models were developed using AP, PP, HBP, and combined phase images. Performance metrics across training, internal, and external test sets are presented in **Supplementary material Table S2**. Both the Habitat and DL models in HBP showed higher AUCs compared with other phase models in the internal test set.

**Figure 2:**
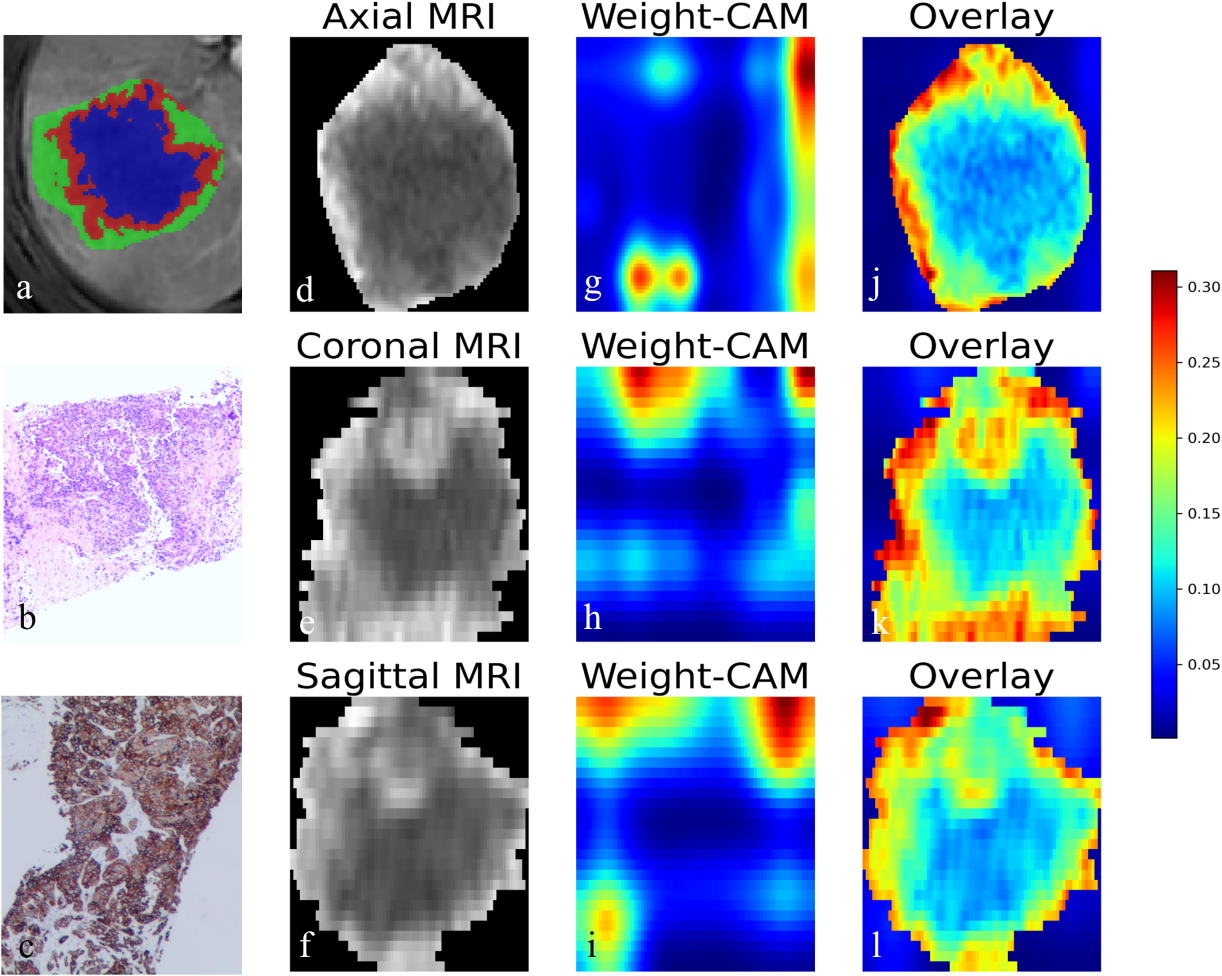
Visualization of habitat and deep learning features in CK19+ HCC. **(a)** Axial MRI image showing the habitat clustering of tumor regions. **(b)** HE staining elucidated the hepatocytic origin of HCC. **(c)** IHC staining showing CK19 positivity. **(d-f)** Axial, coronal, and sagittal MRI views of the tumor. **(g-i)** Corresponding Class Activation Maps (CAM) from deep learning model showing attention weights. **(j-l)** Overlay of CAM weights on the MRI images, where the red color highlighted that model focuses attention on the tumor periphery. HCC, Hepatocellular carcinoma. HE, hematoxylin-eosin; IHC, immunohistochemistry.

In the external test set, the AUCs of the Habitat model were 0.590 [95% CI: 0.485-0.696], 0.547 [95% CI: 0.426-0.667], 0.715 [95% CI: 0.603-0.827], and 0.570 [95% CI: 0.461-0.678] for AP, PP, HBP, and combined phase, respectively. The AUCs of the DL model were 0.569 [95% CI: 0.467-0.670], 0.632 [95% CI: 0.526-0.737], 0.581 [95% CI: 0.470-0.697], and 0.584 [95% CI: 0.480-0.689] for AP, PP, HBP, and combined phase, respectively. The visualization of habitat and deep learning features for a CK19+ case is shown in **Figure 2**. The CAM heatmaps highlight that tumor margin regions receive significant attention, corresponding to the habitat marginal regions.

Integration of habitat and deep learning features was subsequently used to develop the Habitat-DL model. (Correlation heatmaps of habitat radiomics and deep learning features in HBP are shown in **Supplementary material Figure S1**). The performance of Habitat-DL model was highest in combined phase, achieving AUCs of 0.832 [95% CI: 0.775-0.888], 0.728 [95% CI: 0.626-0.831], and 0.695 [95% CI: 0.586-0.804] in training, internal, and external test sets, respectively (**Supplementary material Table S3**).

#### 3.2.3 Comparison of performance between the DL-HR nomogram and clinical-radiologic model

Multivariable analysis identified AFP level > 100 μg/L, AP enhancement, and the predictive scores derived from DL and Habitat models as independent predictors of CK19 expression without multicollinearity (VIF <2) (**Supplementary material Table S4**). By integrating clinical, radiologic, habitat radiomics, and deep learning features, the DL-HR nomogram achieved improved AUCs of 0.863 [95% CI: 0.815-0.902], 0.794 [95%CI: 0.708-0.864], and 0.744 [95% CI:0.669-0.810] in the training, internal, and external test sets, respectively. The DL-HR nomogram significantly outperformed the clinical-radiologic model across all sets (**Table 4**), with statistically significant differences in both internal and external test sets. (Delong test, all p < 0.05).

**Figure 3:**
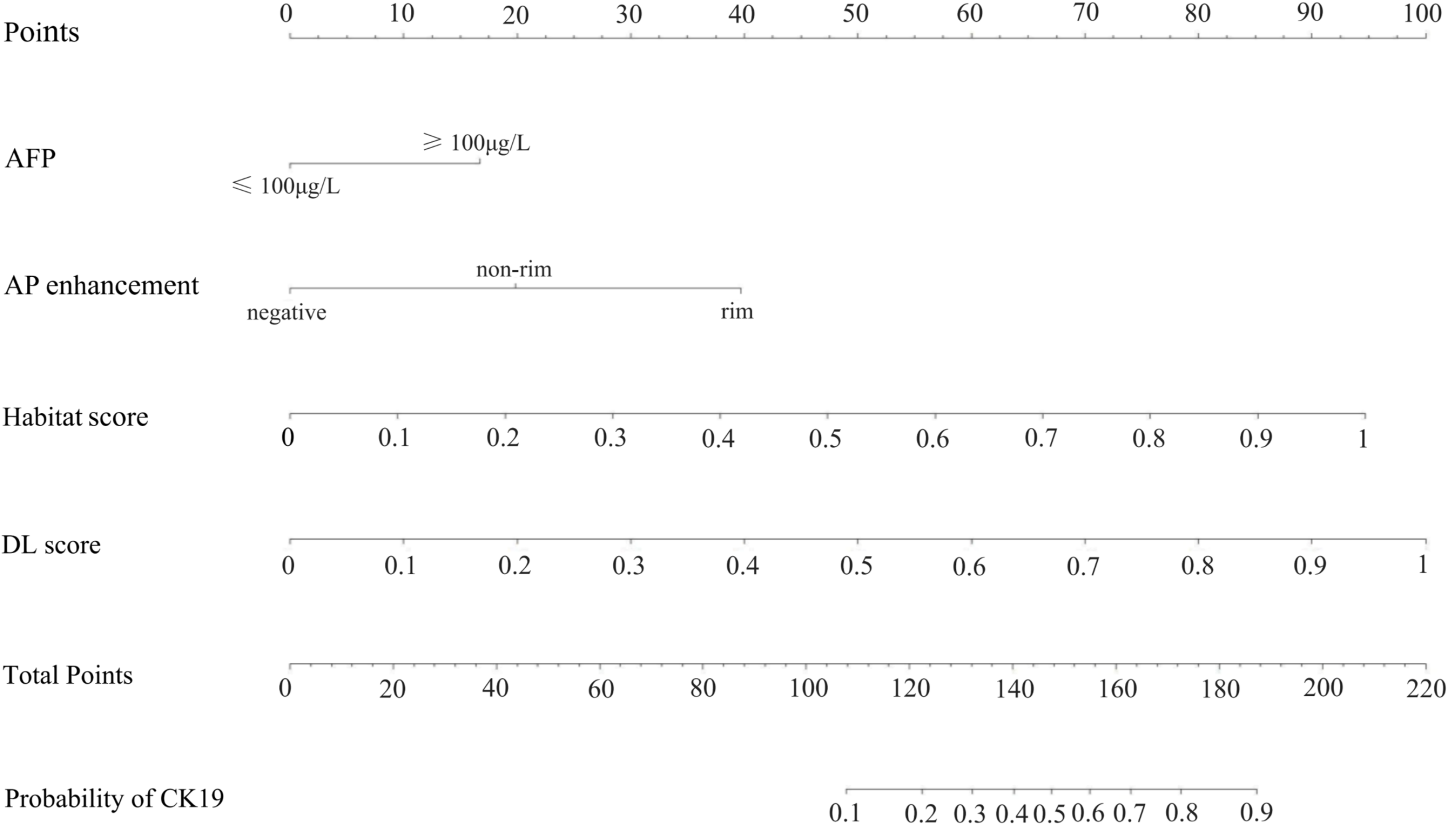
The utilization of the nomogram to predict the CK19 expression. The nomogram incorporates four independent predictors: AFP level, AP enhancement, Habitat model score, and deep learning (DL) model score. AP, arterial phase. DL, deep learning.

**Figure 4:**
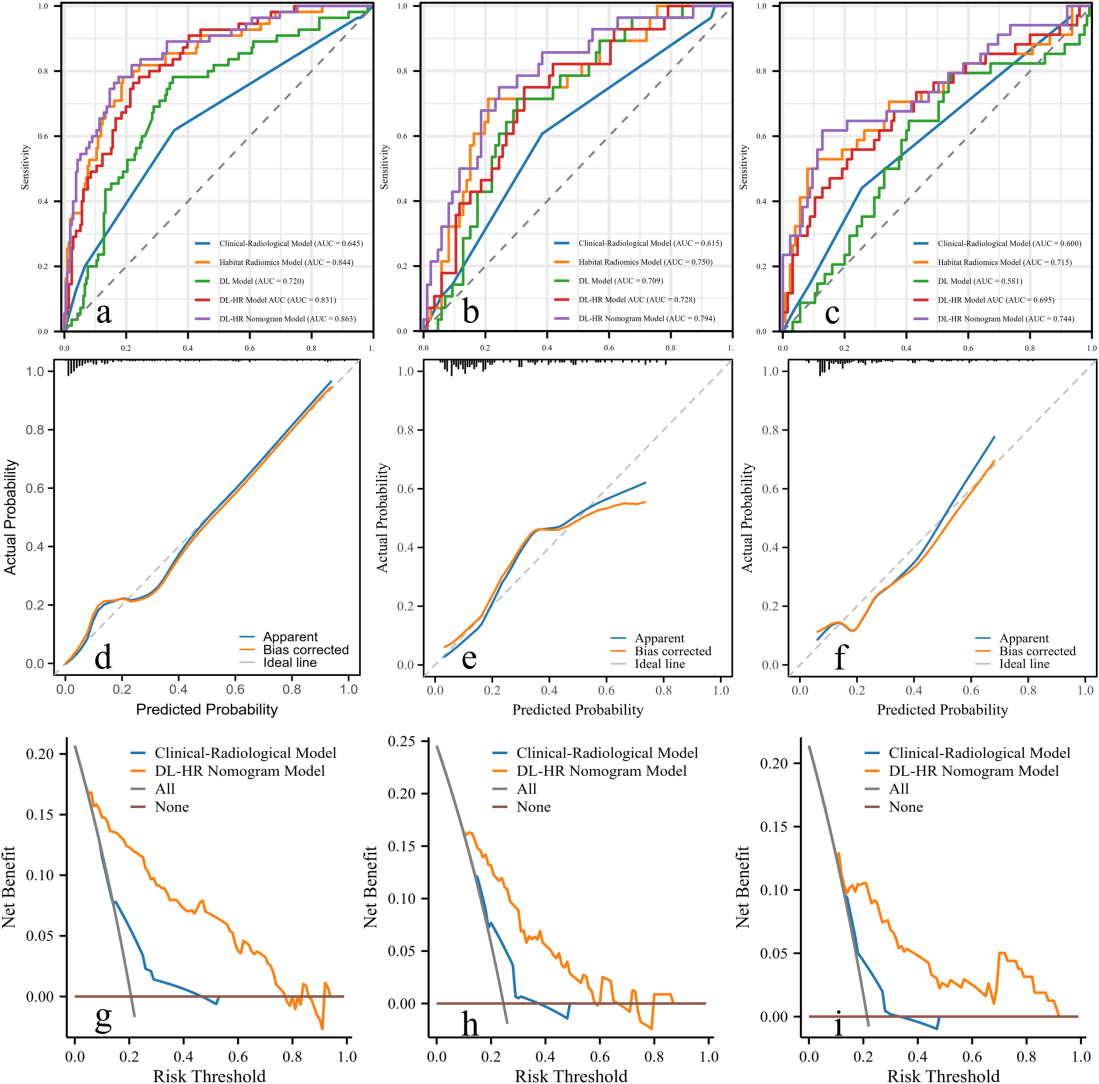
Receiver operating characteristic curves, calibration curves, and decision curve of the DL-HR nomogram model. Five models are displayed: Clinical-Radiological Model (blue), Habitat Model (orange), DL Model (green), DL-HR Model (red), and DL-HR nomogram Model (purple). The DL-HR nomogram Model demonstrated superior performance with the highest AUC values in training **(a)**, internal **(b)**, and external test set **(c)**. The calibration curves exhibited satisfactory concordance between the predicted and observed probabilities of CK19+ HCC in both the training **(d)**, internal **(e)** and external test set **(f)**. The decision curve analysis of the prediction model was performed for the training **(g)**, internal **(h)**, and external test set **(i)**. The curve of the DL-HR nomogram model demonstrated favorable benefits. AUC, area under the curve. ROC, receiver operating characteristic.

**Figure 5:**
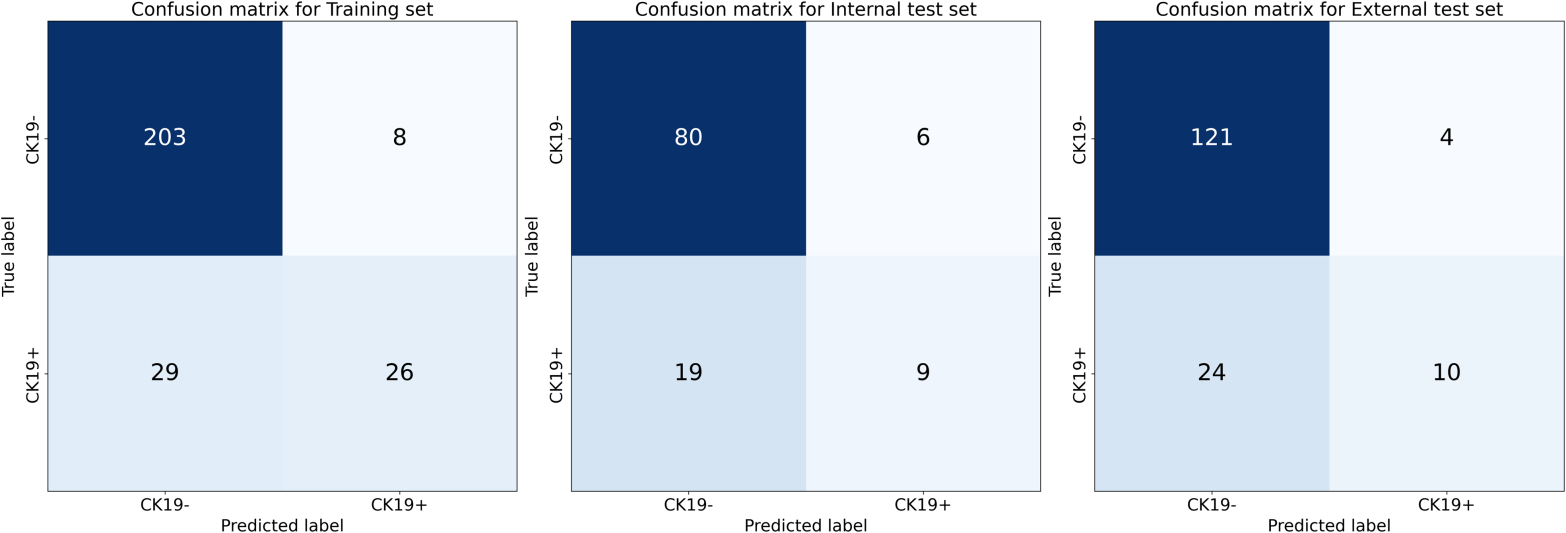
Confusion matrices of DL-HR nomogram model. The matrices display model performance in classifying CK19-negative and CK19+ HCC. Training set: 203 true CK19- cases correctly classified with 8 false positives, and 26 true CK19+ cases correctly classified with 29 false negatives. Internal test set: 80 true CK19- correctly identified with 6 false positives, and 9 true CK19+ correctly identified with 19 false negatives. External test set: 121 true CK19- correctly identified with 4 false positives, and 10 true CK19+ correctly identified with 24 false negatives.

Calibration curves for the DL-HR nomogram model predicted outcomes showed no observable difference from the calibration curves for actual CK19 expression across all sets (Hosmer-Lemeshow test: training set, p = 0.504; internal test set, p = 0.849; external test set, p = 0.588). The nomogram to predict the CK19 expression is shown in **Figure 3**. The evaluation of DL-HR nomogram model is shown in **Figure 4**. Confusion matrices of DL-HR nomogram model are shown in **Figure 5**.

### 3.3 DL-HR nomogram model for RFS

By February 10, 2025, 434 of 539 (80.5%) patients were enrolled for RFS analysis. The overall recurrence rate was 36.7% (159 of 434), with rates of 36.2% (80 of 221), 33.7% (33 of 98), and 40% (46 of 115) in the training, internal, and external test sets. Patients have a median RFS of 32.0 (IQR, 16.0-48.2) months. CK19+ HCC revealed poorer RFS compared with CK19- HCC in the whole set (n = 434). The median RFS was 26.0 [95%CI: 20.5-31.5] months for CK19+ patients and 34.6 [95%CI: 28.1-41.1] months for CK19- patients (p = 0.031).

**Figure 6:**
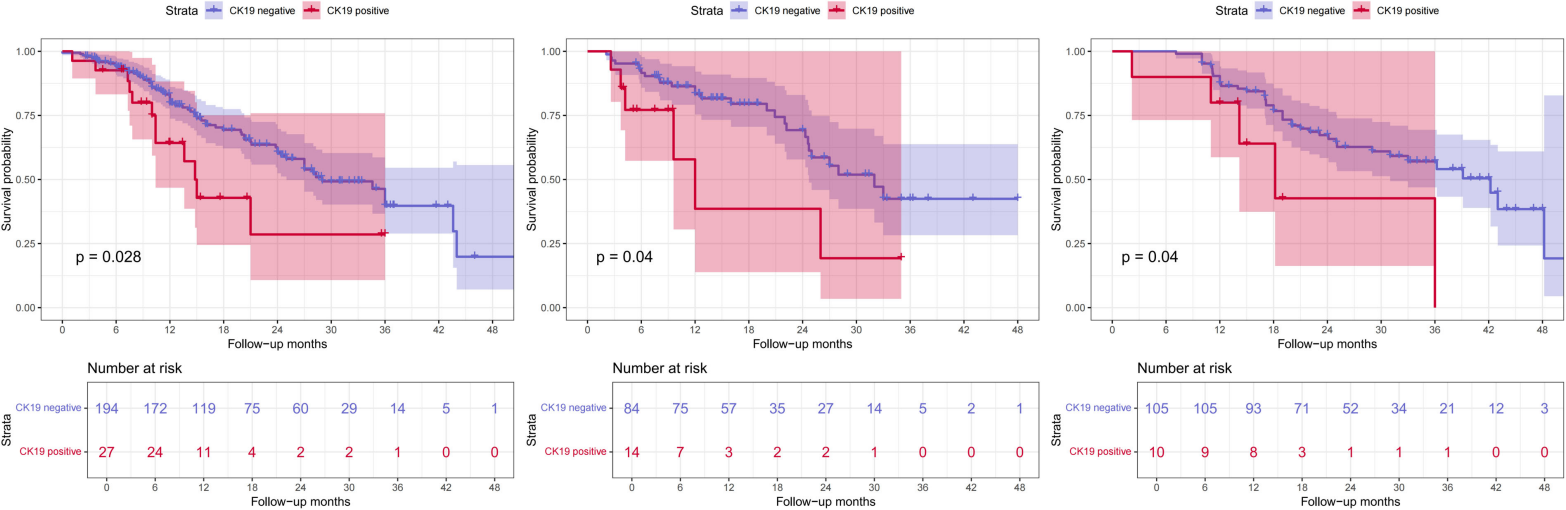
Kaplan-Meier survival curve of recurrence-free survival (RFS). DL-HR nomogram-predicted CK19 expression in training **(a)**, internal **(b)**, and external test sets **(c)**. RFS, recurrence-free survival.

The DL-HR nomogram model had similar results, with patients predicted to be CK19+ exhibiting significantly shorter median RFS than across all sets: training set (14.8 vs. 29.0 months), internal test set (12.0 vs. 32.0 months), and external test set (18.2 vs. 42.2 months). These differences were statistically significant (log-rank test: p = 0.028, 0.040, and 0.040, respectively) (**Figure 6**).

## 4 Discussion

CK19+ HCC is a hyperproliferative subtype of HCC that shows higher AFP levels, poor differentiation, frequent vascular invasion, and worse prognosis (15, 16). Therefore, developing non-invasive methods to identify these hyperproliferative subtypes has significant clinical value. In this multicenter study, we found four independent predictors of CK19 expression: AFP level > 100 μg/L, AP enhancement, and predictive scores derived from both Habitat model and DL models. By integrating these factors, we developed a DL-HR nomogram model showed the highest AUC values in the internal test (AUC, 0.794) and maintained robust performance in the external test set (AUC, 0.744), indicating its potential as a valuable tool for CK19 prediction. Furthermore, the DL-HR nomogram effectively stratified HCC patients into risk categories for RFS across all sets.

Logistic regression analysis showed that elevated AFP level and AP enhancement were independent predictors. These findings support the biological relationship between imaging phenotypes and the molecular characteristics of CK19+ HCC, consistent with previous studies (3). Traditional radiomics analysis typically extracts features from the whole tumor, whereas habitat analysis defines subregional divisions that reflect diverse voxel intensity information to better characterize intratumoral heterogeneity (17, 18). Previous studies have utilized radiomics to predict CK19 expression, such as Fan Yang et al.’s (19) predictive model achieving AUCs of 0.857, 0.726, and 0.790 in training and validation cohorts, respectively. Compared with their studies, our method incorporates more comprehensive enhanced sequence information and employs gadoxetic acid, a liver-specific contrast agent that improves detection and differential diagnosis of liver space-occupying lesions (20, 21). Additionally, combined habitat analysis with deep learning approaches to enhance model performance. While Wang et al.’s fusion radiomics prediction model achieved higher AUCs of 0.951 and 0.822 in training and validation data sets (14), it is important to note that HCC is a heterogeneous disease with substantial variations in outcomes and treatment responses. Information extracted from the entire tumor may introduce significant interference factors, including hemorrhage, necrosis, cystic changes, potentially confounding tumor heterogeneity and reducing predictive power. This study implemented an unsupervised K-means clustering algorithm to generate cluster label maps for intratumoral voxels. These label maps visualized clustering pattern distributions and quantified tumor heterogeneity. Therefore, our Habitat imaging enables meticulous observation of the complex variations within the tumor habitat, improving the ability to capture tumor complexity and better represent actual tumor behavior. We speculate that peripheral subregions may correspond to areas with abundant vascularity and active cellular proliferation. Previous studies have reported that CK19-positive HCC often exhibits arterial-phase rim enhancement. These observations are consistent with our findings and support the association of CK19 positivity with high proliferative activity and poor differentiation. Transitional subregions likely represent mixed cellular areas containing tumor cells, fibrous septa, and other components. Conversely, subregions closer to the tumor center may reflect necrosis or extensive fibrosis, corresponding to hypoxic and necrotic areas within the tumor. In fact, habitat radiomics-based predictive models have demonstrated excellent performance across various cancers, including esophageal cancer, breast cancer, and rectal cancers (22–24).

The deep learning methods in this study utilized a Swin Transformer framework that effectively captures complex spatial relationships through its multi-attention mechanisms, particularly valuable for analyzing image features (25, 26). The self-attention mechanism allows the model to focus on relevant image regions while considering the global context, enhancing feature extraction capabilities beyond conventional CNNs (27, 28). Deep learning models are increasingly applied to diagnose pathological features, predict treatment response, and identify early recurrence in various malignancies (29–31). Previous reports found that deep learning can be applied to the HCC classification and identification (32, 33), as shown by Charlie A. Hamm’s research (34). Compared with Hai-Feng Liu et al.’s study (35), our research similarly integrated habitat and deep learning features. Moreover, we enhanced our model by incorporating comprehensive clinical data and traditional imaging features, increasing its clinical applicability. Furthermore, their study was limited by its single-institution design, relatively modest sample size, and lacked external validation cohorts. Indeed, the integration of diverse features to create optimized models has been substantiated across multiple studies (36–38).

This study has several limitations. First, this is a retrospective study, which may introduce inherent biases, including potential selection bias reflected in the predominance of male participants, although this gender distribution aligns with the higher incidence of primary liver cancer in East Asian males (39). Second, despite the substantial sample size (n=539), it remains relatively modest for deep learning applications. Third, variations in imaging equipment and baseline characteristics across two institutions may affect deep learning and habitat features. Finally, all tumor images were manually outlined by radiologists and future research should investigate automatic lesion segmentation to improve the stability of our model.

In conclusion, we developed and validated a DL-HR nomogram model combining clinical, radiologic, habitat radiomics, and deep learning features for the prediction of CK19 expression in HCC. Furthermore, the model effectively stratified patients into different risk categories for RFS, highlighting its potential clinical utility for treatment planning and post-operative surveillance. Future prospective studies are needed to validate the clinical applicability of this model.

## Data Availability

The raw data supporting the conclusions of this article will be made available by the authors, without undue reservation.

## Supplementary material

Supplementary Figure 1 Correlation heatmaps of habitat radiomics and deep learning features in HBP.
