# Supplementary figures and images for: Habitat radiomics and deep learning on gadoxetic acid-enhanced MRI for noninvasive assessment of CK19 expression and recurrence-free survival in hepatocellular carcinoma
Source: Front Oncol. 2025 Nov 10;15:1684264. doi: 10.3389/fonc.2025.1684264 (PMC12641396; doi:10.3389/fonc.2025.1684264)

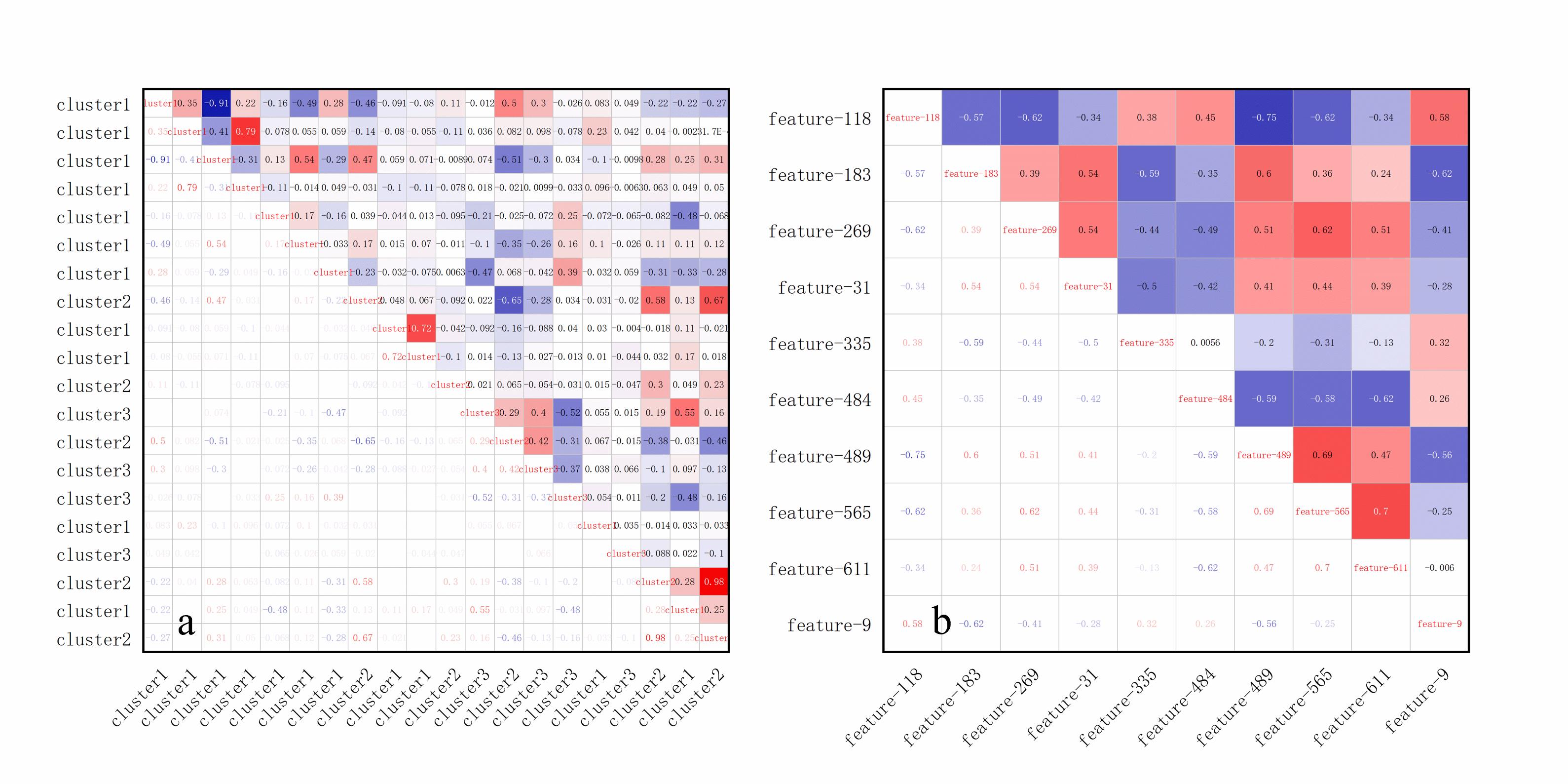

Supplement: Supplementary Figure 1 — Correlation heatmaps of habitat radiomics and deep learning features in HBP. [file Image1.tif]

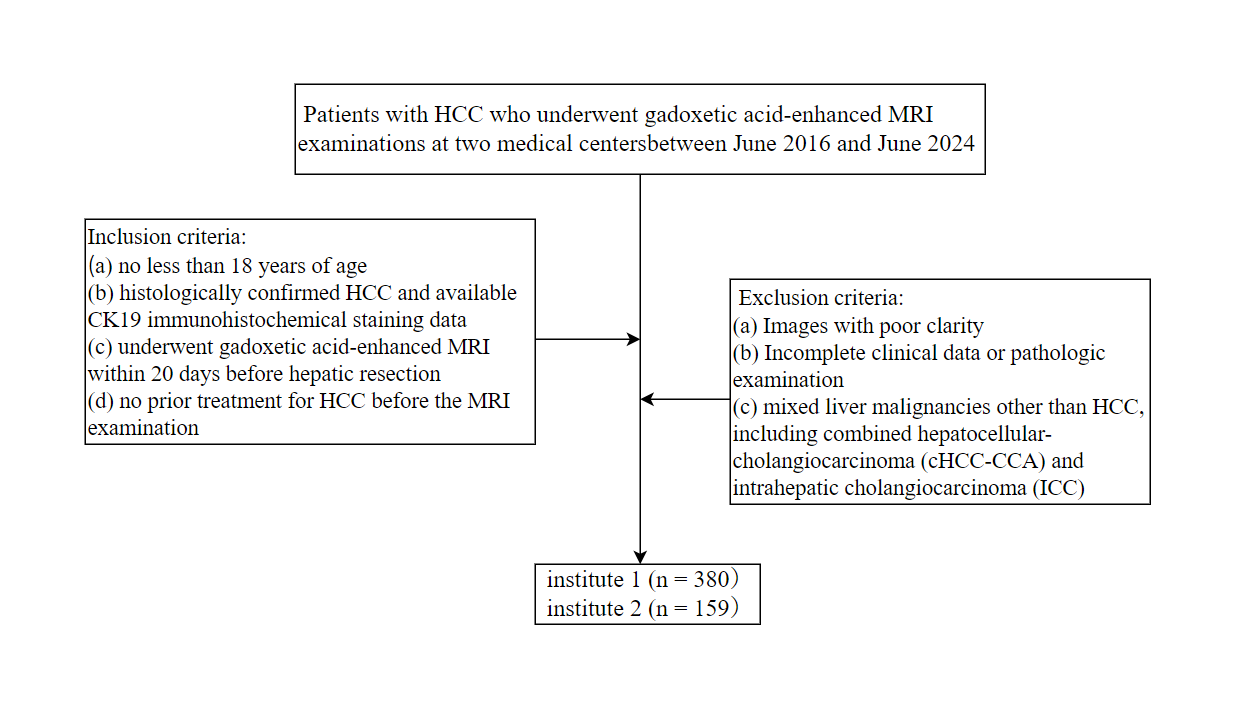

Supplement: Supplementary file 2 [file Image2.png]
