# Supplementary material for: Habitat radiomics and deep learning on gadoxetic acid-enhanced MRI for noninvasive assessment of CK19 expression and recurrence-free survival in hepatocellular carcinoma
Source: Front Oncol. 2025 Nov 10;15:1684264. doi: 10.3389/fonc.2025.1684264 (PMC12641396; doi:10.3389/fonc.2025.1684264)
Supplement: Supplementary file 3 [file Table1.docx]

**Supplementary material**

**Table S1 MRI acquisition**

The MRI scan sequences and parameters (Siemens Magnetom Skyra3.0T)

| Sequence | Category | TR (ms) | TE (ms) | FOV  (mm) | Thickness  (mm) | Fat saturation | Breath-hold |
| --- | --- | --- | --- | --- | --- | --- | --- |
| T1WI in/ opposed phase | 3D VIBE | 4.11 | 2.47/1.24 | 400🞩400 | 3.5 | No | Yes |
| T1WI-fs | 3D VIBE | 4.11 | 1.24 | 400🞩400 | 3.5 | Yes | Yes |
| T2WI-fs | TSE | 3000 | 82 | 400🞩400 | 6.5 | Yes | Yes |
| DWI | Ep2d | 5600 | 58 | 400🞩400 | 6.5 | Yes | No |
| Contrast-enhanced |  |  |  |  |  |  |  |
| T1WI-fs （AP） | 3D VIBE | 4.15 | 2.01 | 400🞩400 | 3.5 | Yes | Yes |
| T1WI-fs （PP） | 3D VIBE | 4.15 | 2.01 | 400🞩400 | 3.5 | Yes | Yes |

Notes: FOV: field of view; 3D VIBE, a three-dimensional volume interpolated breath-hold examination; Ep2d, a two-dimensional echo-planar technique; fs, fat suppression; TSE, turbo spin-echo; HBP, hepatobiliary phase.

The MRI scan sequences and parameters (GE Revolution-3.0T)

| Sequence | Category | TR (ms) | TE (ms) | FOV  (mm) | Thickness  (mm) | Fat saturation | Breath-hold |
| --- | --- | --- | --- | --- | --- | --- | --- |
| T1WI in/ opposed phase | Fast SPGR | 4.6 | 1.2 | 400🞩360 | 3 | No | Yes |
| T1WI-fs | LAVA-Flex | 4.5 | 1.4 | 380🞩342 | 2.5 | Yes | Yes |
| T2WI-fs | FRFSE-XL | 7500 | 86.2 | 400🞩300 | 2 | Yes | Yes |
| DWI | DW EPI | 10000 | 57.1 | 400🞩320 | 2 | Yes | No |
| Contrast-enhanced |  |  |  |  |  |  |  |
| T1WI-fs (AP) | LAVA-Flex | 4.6 | 1.7 | 380🞩342 | 2.5 | Yes | Yes |
| T1WI-fs (PP) | LAVA-Flex | 4.6 | 1.7 | 380🞩342 | 2.5 | Yes | Yes |

Notes: FOV: field of view; 3D VIBE, a three-dimensional volume interpolated breath-hold examination; Ep2d, a two-dimensional echo-planar technique; fs, fat suppression; TSE, turbo spin-echo; HBP, hepatobiliary phase.

**Table S2 Performance of Habitat-DL Models**

| Models | AUC (95%CI) | Sensitivity | Specificity | MCC |
| --- | --- | --- | --- | --- |
| Habitat Model |  |  |  |  |
| AP |  |  |  |  |
| train | 0.800(0.739-0.859) | 0.873 | 0.645 | 0.420 |
| Internal test | 0.540(0.409-0.670) | 0.464 | 0.709 | 0.159 |
| External test | 0.590(0.485-0.696) | 0.882 | 0.320 | 0.186 |
| PP |  |  |  |  |
| train | 0.818(0.757-0.878) | 0.746 | 0.787 | 0.461 |
| Internal test | 0.547(0.412-0.682) | 0.429 | 0.791 | 0.214 |
| External test | 0.547(0.426-0.667) | 0.500 | 0.672 | 0.147 |
| HBP |  |  |  |  |
| train | 0.844(0.785-0.903) | 0.782 | 0.810 | 0.518 |
| Internal test | 0.750(0.647-0.853) | 0.714 | 0.791 | 0.461 |
| External test | 0.715(0.603-0.827) | 0.500 | 0.920 | 0.459 |
| Combined phase |  |  |  |  |
| train | 0.806(0.741-0.872) | 0.673 | 0.844 | 0.475 |
| Internal test | 0.740(0.633-0.846) | 0.643 | 0.802 | 0.416 |
| External test | 0.570(0.461-0.678) | 0.588 | 0.632 | 0.183 |
| DL Model |  |  |  |  |
| AP |  |  |  |  |
| train | 0.735(0.663-0.806) | 0.855 | 0.517 | 0.303 |
| Internal test | 0.493(0.368-0.619) | 0.286 | 0.802 | 0.092 |
| External test | 0.569(0.467-0.670) | 0.735 | 0.448 | 0.153 |
| PP |  |  |  |  |
| train | 0.709(0.638-0.780) | 0.873 | 0.446 | 0.266 |
| Internal test | 0.558(0.435-0.681) | 0.500 | 0.663 | 0.145 |
| External test | 0.632(0.526-0.737) | 0.677 | 0.536 | 0.174 |
| HBP |  |  |  |  |
| train | 0.720(0.645-0.794) | 0.7818 | 0.6493 | 0.352 |
| Internal test | 0.709(0.607-0.812) | 0.7143 | 0.6977 | 0.3615 |
| External test | 0.581(0.470-0.697) | 0.7941 | 0.456 | 0.2091 |
| Combined phase |  |  |  |  |
| train | 0.727(0.654-0.800) | 0.7273 | 0.6493 | 0.3082 |
| Internal test | 0.591(0.464-0.719) | 0.5714 | 0.7093 | 0.2518 |
| External test | 0.584(0.480-0.689) | 0.7353 | 0.424 | 0.1339 |

Note: Sensitivity and specificity are percentages, 95% CI = 95% confidence interval, MCC = matthews correlation coefficient, AP = arterial phase, PP = portal venous phase, HBP = hepatobiliary phase, AUC = area under the receiver operating characteristic curve, DL = deep learning; Swin Transformer was developed with the Swin_v2_b model in the torchvision library in PyTorch.

**Table S3 Performance of Habitat-DL Models**

| Habitat-DL Model | AUC (95%CI) | Sensitivity | Specificity | MCC |
| --- | --- | --- | --- | --- |
| AP |  |  |  |  |
| train | 0.781(0.716-0.846) | 0.618 | 0.859 | 0.451 |
| Internal test | 0.574(0.444-0.705) | 0.571 | 0.640 | 0.185 |
| External test | 0.584(0.473-0.694) | 0.471 | 0.704 | 0.152 |
| PP |  |  |  |  |
| train | 0.738(0.665-0.810) | 0.746 | 0.673 | 0.344 |
| Internal test | 0.657(0.536-0.778) | 0.643 | 0.651 | 0.256 |
| External test | 0.651(0.546-0.755) | 0.647 | 0.608 | 0.210 |
| HBP |  |  |  |  |
| train | 0.782(0.710-0.854) | 0.709 | 0.758 | 0.400 |
| Internal test | 0.660(0.544-0.776) | 0.560 | 0.719 | 0.243 |
| External test | 0.673(0.567-0.778) | 0.853 | 0.448 | 0.254 |
| Combined phase |  |  |  |  |
| train | 0.832(0.775-0.888) | 0.764 | 0.777 | 0.464 |
| Internal test | 0.728(0.626-0.831) | 0.750 | 0.674 | 0.369 |
| External test | 0.695(0.586-0.804) | 0.559 | 0.776 | 0.301 |

**Note: Sensitivity and specificity are percentages, 95% CI = 95% confidence interval, MCC = matthews correlation coefficient, AP = arterial phase, PP = portal venous phase, HBP = hepatobiliary phase, AUC = area under the receiver operating characteristic curve.**

**Table S4 The predictors with collinearity analysis**

|  | Β | t | P | VIF |
| --- | --- | --- | --- | --- |
| Age | -0.002 | -0.926 | 0.355 | 1.123 |
| AFP | 0.094 | 2.154 | 0.032 | 1.105 |
| AP hyper enhancement | 0.099 | 1.764 | -0.012 | 1.071 |
| Washout | 0.035 | 0.894 | -0.042 | 1.071 |
| HBP peritumoral hypointensity | 0.007 | 0.133 | -0.101 | 1.134 |
| Habitat score | 0.636 | 9.305 | 0.502 | 1.052 |
| DL score | 0.540 | 5.184 | 0.335 | 1.009 |

Note:VIF = Variance Inflation Factor.
